# Transcriptome profile in *Drosophila* Kc and S2 embryonic cell lines

**DOI:** 10.1093/g3journal/jkad054

**Published:** 2023-03-03

**Authors:** Daniel Klonaros, Jacqueline M Dresch, Robert A Drewell

**Affiliations:** Biology Department, Clark University, 950 Main Street, Worcester, MA 01610, USA; Biology Department, Clark University, 950 Main Street, Worcester, MA 01610, USA; Biology Department, Clark University, 950 Main Street, Worcester, MA 01610, USA

**Keywords:** *Drosophila*, embryo, cell lines, S2, Kc, transcriptome

## Abstract

*Drosophila melanogaster* cell lines are an important resource for a range of studies spanning genomics, molecular genetics, and cell biology. Amongst these valuable lines are Kc167 (Kc) and Schneider 2 (S2) cells, which were originally isolated in the late 1960s from embryonic sources and have been used extensively to investigate a broad spectrum of biological activities including cell–cell signaling and immune system function. Whole-genome tiling microarray analysis of total RNA from these two cell types was performed as part of the modENCODE project over a decade ago and revealed that they share a number of gene expression features. Here, we expand on these earlier studies by using deep-coverage RNA-sequencing approaches to investigate the transcriptional profile in Kc and S2 cells in detail. Comparison of the transcriptomes reveals that ∼75% of the 13,919 annotated genes are expressed at a detectable level in at least one of the cell lines, with the majority of these genes expressed at high levels in both cell lines. Despite the overall similarity of the transcriptional landscape in the two cell types, 2,588 differentially expressed genes are identified. Many of the genes with the largest fold change are known only by their “CG” designations, indicating that the molecular control of Kc and S2 cell identity may be regulated in part by a cohort of relatively uncharacterized genes. Our data also indicate that both cell lines have distinct hemocyte-like identities, but share active signaling pathways and express a number of genes in the network responsible for dorsal–ventral patterning of the early embryo.

## Introduction

The first embryonic *Drosophila melanogaster* cell lines were established in the late 1960s. Two of the most widely studied lines are Kc167 (Echalier and Ohanessian 1969) and Schneider 2 (Schneider 1972) cells, both of which were originally isolated from fruit fly embryos. While the exact history of these cell lines is not entirely documented, there is evidence that Kc167 (Kc) cells were isolated from embryos at stage 13–15 (dorsal closure) and have plasmatocyte-like properties (Cherbas *et al*. 1988; Andres and Cherbas 1992), while Schneider 2 (S2) cells appear to originate from embryos at stage 16–17 (late embryonic) (Schneider 1972). Both cell lines have been extensively used in wide-ranging studies of biological processes including cell–cell signaling, hormone responses, heat shock, and immune system function (Luhur *et al*. 2019). More recently, these cells have been instrumental in developing insightful RNAi-based screens (Bakal and Perrimon 2010), CRISPR-based functional genomics (Mohr *et al*. 2014; Housden *et al*. 2015), and models of viral infection (Zhu *et al*. 2013; Merkling *et al*. 2015).

A whole-genome tiling microarray analysis of total RNA from both cell lines was included as part of the modENCODE project (Celniker *et al*. 2009; Roy *et al*. 2010) to characterize the transcriptional diversity of 25 different *Drosophila* cell lines (Cherbas *et al*. 2011). On average, expression was detectable for 5,885 genes in each cell line, with a common set of 3,109 (representing 21% of the 14,807 genes probed) expressed in all lines (Cherbas *et al*. 2011). Principal component analysis revealed that while each of the 25 cell lines has a distinct expression profile, there is a coherent trajectory of changing gene expression patterns that correlates with the reported embryonic, larval or pupal stage from which the cells were originally isolated (Cherbas *et al*. 2011; Graveley *et al*. 2011). The authors note that there is a tight clustering of all cells, including the Kc and S2 lines, near the expression profile of early embryos (Cherbas *et al*. 2011). The Kc and S2 cells also display evidence of a hematopoietic origin based on their respective gene expression patterns. Specifically, the data support a plasmatocyte identity for the Kc cell line and suggest a somewhat more plastic hemocyte identity for the S2 cell line (Cherbas *et al*. 2011).

In this current work, we expand on these earlier studies by using cutting-edge RNA-sequencing approaches to investigate the transcriptome in Kc and S2 cells (see Table 1). Deep-coverage sequencing enables us to compare the transcriptional profile of the two cell lines in detail and highlights some key shared features and differences between the two embryonic cell types. This data contributes to our understanding of the cell lines and further opens up the possibility of using these cells to investigate critical components of the molecular genetic control of events in embryonic development.

**Table 1.** Embryonic cell lines analyzed in this study.

Cell line	Short name	Reference	Clean Reads(M)	Mapping rate (%)
Kc-167	KC	Echalier and Ohanessian 1969	45.35	77.74
S2-DRSC	S2	Schneider 1972	45.48	76.94

The two cell lines and their respective total reads and mapping rate are indicated.

## Methods

### Cell culture

Cells were obtained from the Drosophila Genomics Resource Center (DGRC). The Kc167 (Kc, RRID: CVCL_Z833) and S2-DRSC (S2, RRID: CVCL_Z992) cell lines used in this study are listed in Table 1. Cells were thawed, passaged, and frozen according to DGRC protocols (https://dgrc.bio.indiana.edu/Protocols? tab = cells). Cells were maintained between ∼2 × 10^6^ and 1 × 10^7^ cells/mL at 25 °C in Schneider's *Drosophila* medium (Gibco) supplemented with 10% fetal bovine serum (FBS, Gibco) and penicillin–streptomycin (Gibco) at a final concentration of 100 U/ml. To minimize potential contamination, all cells were grown in an isolated tissue culture room, which included a HEPA-filtered Class II biosafety cabinet and HEPA-filtered incubator.

### RNA isolation

Total RNA was isolated as previously described (Cherbas *et al*. 2011) from six replicate cell samples grown in 25 cm^2^ canted neck culture flasks (Corning). Cells were harvested at ∼5 × 10^6^ cells/mL density. After centrifugation, the RNA was pooled and extracted using a RNeasy kit following the manufacturer's protocol (Qiagen) and dissolved in nuclease-free water. Concentration was determined by absorbance using a Nanodrop spectrophotometer. Preliminary quality of each RNA sample was analyzed by gel electrophoresis. All samples were stored at −80 °C and shipped on dry ice using overnight delivery.

### RNA sequencing

Library construction and sequencing were performed at the Beijing Genomics Institute. Briefly, 10µg of total RNA was enriched for poly(A)^+^ RNA by oligo(dT) selection. The poly(A)^+^ RNA was then fragmented and first-strand cDNA synthesis was performed using random N6-primed reverse transcription, followed by second-strand synthesis with dUTP. The synthesized cDNA was subjected to end-repair, then 3′ adenylated and adaptors were ligated to the ends of these fragments. Prior to PCR amplification, the dUTP-marked strand was selectively degraded by Uracil-DNA-Glycosylase (UDG). The remaining strand was amplified to generate a cDNA library for sequencing. The libraries were enriched by multiple rounds of PCR amplification to enrich the purified cDNA template. The libraries were used for sequencing on the Illumina nanoball (DNBSEQ) PE100 platform.

Sequencing data was filtered using SOAPnuke software v1.5.2 (https://github.com/BGI-flexlab/SOAPnuke) to remove reads containing the adaptor sequences, reads whose N content was greater than 5%, and low-quality reads (quality score less than 15 for 20% or greater of the total bases in the given read). The generated clean read fastq files (Cock *et al*. 2010) were aligned using Bowtie2 software (Langmead and Salzberg 2012) to the *Drosophila melanogaster* genome (Release 6 plus ISO1 mitochondrial, RefSeq accession: GCF_000001215.4). Aligned data were used to calculate quantitative RPKM (Mortazavi *et al*. 2008), FPKM and TPM scores as previously described (Li and Dewey 2011; Wagner *et al*. 2012).

### Expression analysis

Clean reads were mapped to the reference *D. melanogaster* genome using Bowtie2 v2.2.5 (Langmead and Salzberg 2012) and then RSEM v1.2.8 was used to calculate gene expression levels (Li and Dewey 2011). Differentially expressed genes were defined as genes with a False Discovery Rate (FDR) equal to or less than 0.001 and fold change equal to or greater than 2. The R package *pheatmap* was used to perform hierarchical clustering analysis on the set of differentially expressed genes. GO enrichment analysis was performed using the *phyper* R package (https://stat.ethz.ch/R-manual/R-devel/library/stats/html/Hypergeometric.html) in combination with the *qvalue* Bioconductor package (https://bioconductor.org/packages/release/bioc/html/qvalue.html). The *rMATS* statistical model was employed to quantify alternative splicing events (Shen *et al*. 2014) and the Genome Analysis Toolkit (GATK) was utilized to detect SNP and InDel information (McKenna *et al*. 2010). Hematopoietic marker genes and signaling pathway ligand and receptor genes were selected for detailed analysis based on prior expression studies in *Drosophila* cell lines (Cherbas *et al*. 2011). Embryonic genes for expression analysis were selected based on their well-characterized roles in the control of embryonic development (Wolpert *et al*. 2019).

## Results

### Overview of expression data

**Fig. 1.**
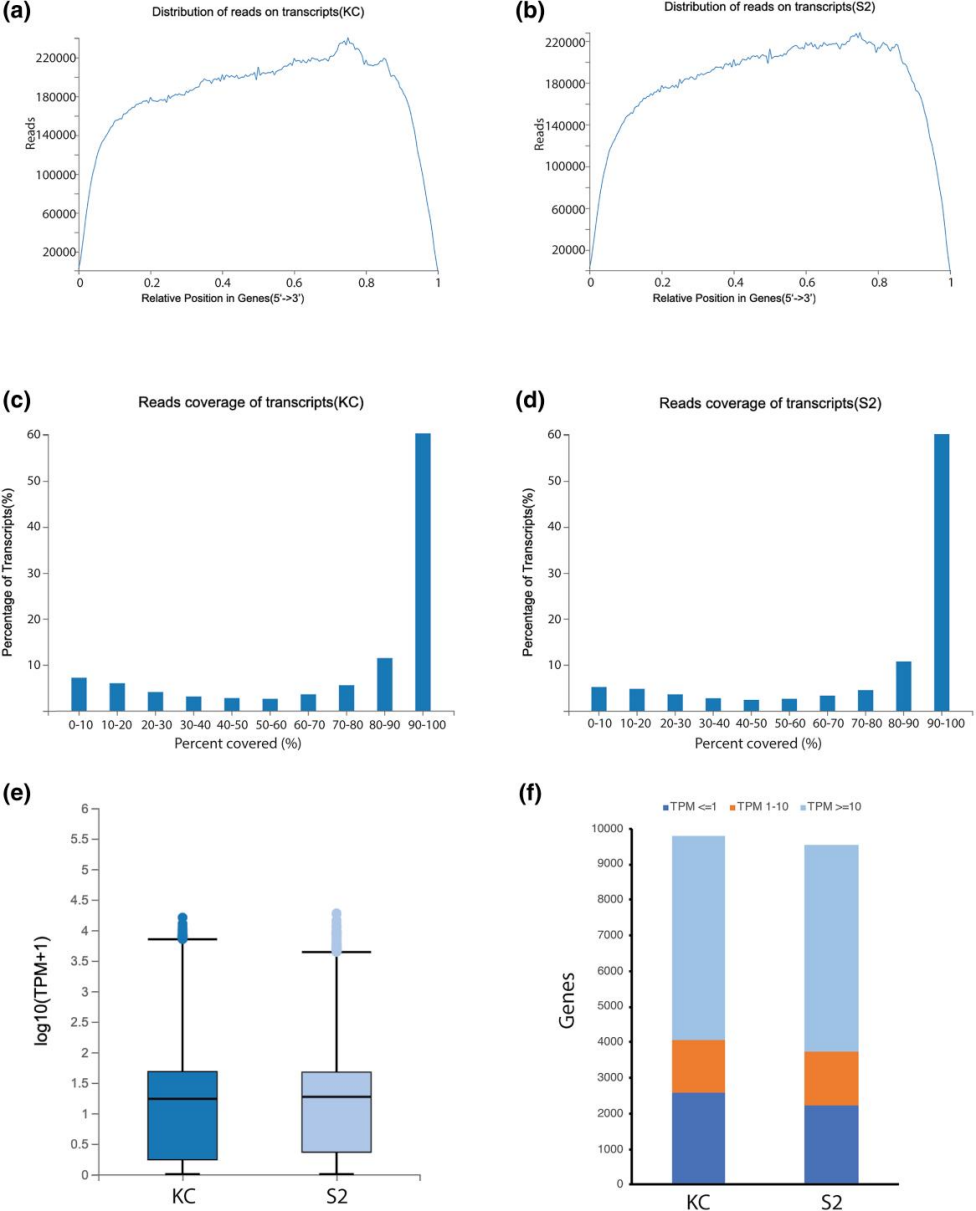
Sequencing alignment, coverage and quality. Distribution of sequencing reads on annotated transcripts in Kc a) and S2 b) cells in a sliding 200 bp window. The relatively even distribution of the reads across the transcripts indicates a sufficient read depth coverage of the transcriptome was achieved. Read coverage of transcripts in Kc c) and S2 d) cells. The coverage at individual transcripts is calculated and organized in 10% bins (i.e. 0–10%, 90–100%) and demonstrate that more than 55% of all the mapped transcripts in each cell type have 90–100% coverage. e) Boxblot indicating maximum, upper quartile, median, lower quartile and minimum TPM expression (log_10_ scale) in Kc (dark blue) and S2 (light blue) cells. The median value in Kc cells is 1.238 and in S2 cells is 1.271. Outlying data points are indicated with individual dots. f) The number of genes with TPM scores <=1 (dark blue), 1–10 (orange) and >=10 (dark blue) in Kc and S2 cells indicate that the overall quantitative expression profile in both cell types is similar.

Samples of poly(A)^+^ RNA were prepared from healthy, exponentially growing Kc167 (Kc) and S2-DRSC (S2) cells (Table 1). Sequencing of the RNA samples on the Illumina nanoball (DNBSEQ) platform generated a total of 90.83 million reads after quality control (see Methods for details), with nearly identical sequencing depth for each cell type (Table 1). Of the sequencing reads, 77.74% from Kc cells and 76.94% from S2 cells mapped unambiguously to the reference *D. melanogaster* genome (Table 1). Both samples also shared a very similar sequencing quality profile, with a relatively even distribution of reads across the body of the mapped transcripts (Fig. 1, a and b) and more than 55% of mapped transcripts with greater than 90% coverage (Fig. 1, c and d). The Pearson correlation coefficient of the overall gene expression profile between with the two samples was 0.894, with a similar distribution of expression quantification as measured by transcripts per million (TPM) metrics (Fig. 1, e and f).

Expression was detected in at least one of the two cell line transcriptomes for 20,731 distinct mRNA transcripts, which equals 68.02% of the 30,480 total annotated mRNA transcripts in the genome (Supplementary Table 1). These mRNAs map to 10,554 (75.82%) of the 13,919 total annotated genes in the genome (Supplementary Table 2). In Kc cells, there was detectable expression of 17,913 distinct mRNAs (58.77% of total) from 9,797 different genes (70.39% of total). In S2 cells, there was detectable expression of 17,896 distinct mRNAs (58.71% of total) from 9,525 genes (68.43% of total). In both cells types, a minority of genes are expressed at very low (TPM <1) or low (TPM 1–10) levels (Fig. 1f). In Kc cells, 4,066 genes are expressed at low levels (representing 41.50% of all the expressed genes), with 2,596 of those (26.50% of all) at very low levels. In S2 cells, 3,728 genes are expressed at low levels (representing 39.14% of all the expressed genes), with 2,234 of those genes (23.45% of all) at very low levels. However, in both cell lines, more than half of all genes with detectable expression have a relatively high TPM >10 (5,731 genes (58.50% of all) in Kc and 5,797 genes (60.86% of all) in S2 cells) (Fig. 1f). Only 985 of the genes are expressed only in Kc cells (Supplementary Table 3) and 723 of the genes are expressed only in S2 cells (Supplementary Table 4), with 90.66% in KC and 83.26% in S2 of these genes, respectively, showing very low-level expression in the cell type in which they are detected.

Transcript expression in both cell lines was exponentially distributed, varying from undetectable to 16,165 in Kc cells and undetectable to 18,989 in S2 cells, with the vast majority of genes expressed at the lower end of this range. These overall distribution profiles are consistent with earlier studies (Cherbas *et al*. 2011). Analysis of *Eip71CD* and *Actin5C* transcripts as a measure of detection sensitivity in our data, reveals a potentially increased sensitivity when compared to prior studies. Saturation hybridization experiments in Kc cells showed the presence of 200–300 *Eip71CD* transcripts per cell, with *Actin5C* giving a signal 5 to 10 times stronger on Northern blots (Bieber 1986). Microarray analysis detected a 3.45-fold expression difference between these two genes in Kc cells (Cherbas *et al*. 2011). In contrast, our RNA-seq data detects a 24.44 fold difference in Kc cells (312.97 *Eip71CD*, 7649.81 *Actin5C*) and 200.85 fold difference in S2 cells (24.31 *Eip71CD*, 4882.75 *Actin5C*), indicating an increased detection sensitivity range in our study.

### Highly expressed genes

We analyzed the expression of 11 genes previously shown to have enhanced and ubiquitous expression in 25 different *Drosophila* cell lines (Cherbas *et al*. 2011). Reassuringly, all 11 of these genes demonstrated high levels of expression in both Kc and S2 cells in our study, with no significant differences in the levels of expression between the two cell types for 9 of the 11 genes (Table 2). There is also extensive overlap in the type of genes with the highest level of expression in Kc (top 20 shown in Supplementary Table 5) and S2 (top 20 shown in Supplementary Table 6) cells. These genes include *eukaryotic translation elongation factor 1 alpha 1* (*eF1alpha1*) and many ribosomal protein encoding (*RpL* and *RpS*) genes, all of which encode for well-studied proteins involved in classic cell housekeeping functions. One notable difference between the two cell types is the very high level of expression of *Neuropeptide-like precursor 2* (*Nplp2*) in S2 cells. The neuropeptide product of this gene is widely expressed in many *Drosophila* cell/tissue types, including hemolymph, and is involved in a number of biological activities, including lipid transport, heat acclimatization and humoral immune response (Rommelaere *et al*. 2019).

**Table 2.** Genes with high level expression in both cell lines.

Gene	Symbol	Gene ID	KC TPM	S2 TPM	KC Read Count	S2 Read Count	log2 (KC/S2)
Karl	Karl	32131	843.11	856.99	13,989	14,373	−0.05
Arc2	Arc2	36597	59.87	45.38	703	536	0.37
Sprouty	Sty	38424	598.95	398.65	29,113	20,777	0.56
CG14696		41317	140.49	87.25	3,118	1,950	0.66
CG15784		31461	1423.72	429.21	38,116	11,559	1.70
BM-40-SPARC	SPARC	43230	1149.62	4009.31	17,412	61,117	−1.83
CG13751		35879	96.66	131.03	419	569	−0.47
Prolyl-4-hydroxylase-alpha EFB	PH4alphaEFB	43620	140.18	110.55	4,311	3,425	0.32
Kekkon-1	kek1	34688	51.54	49.02	4,154	3,733	0.05
Pointed	Pnt	42757	231.82	160.11	10,660	8,407	0.51
Laminin B1	LanB1	34068	342.96	451.42	29,276	38,369	−0.42

The 11 genes listed here were characterized has having enhanced expression in all 25 cell lines analyzed in a previous study using microarray expression data (Cherbas *et al*. 2011). The read counts and corresponding calculated TPM scores for these genes confirm that all 11 are highly expressed in the Kc and S2 cells in our current study, with no significant difference in the level of expression between the two cell types for 9 of the 11 genes (indicated by a log_2_ ratio between −1 and 1).

### Differentially expressed genes

**Fig. 2.**
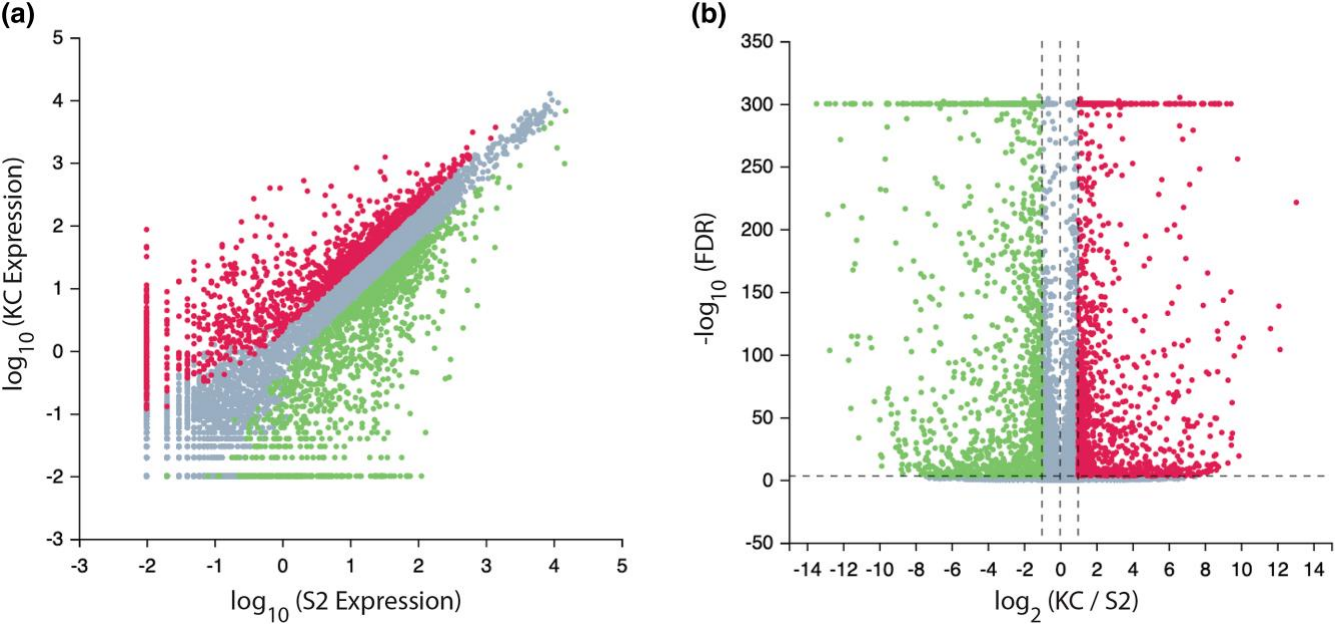
Differentially expressed genes. a) Scatter plot of gene expression level on a log_10_ scale in Kc and S2 cells. b) Volcano plot of expression change ratio (log_2_ Kc/S2) plotted against False Discovery Rate (FDR, -log_10_). While the vast majority of genes are not differentially expressed in the two cell types (grey data points), 1,272 genes are expressed at significantly higher levels in Kc cells than S2 cells (Kc up, red data points) and 1,316 are expressed at lower levels in Kc cells than S2 cells (Kc down, green data points).

Despite the extensive overlap in the overall expression profile between Kc and S2 cells, there are 2,588 detectable differentially expressed genes (DEGs, full list in Supplementary Table 7) with at least a two-fold change in expression between the two cells types (see Methods for full details). Of these DEGs, 1,272 are expressed at relatively higher levels in Kc cells than S2 cells (Kc up, shown in red on Fig. 2) and 1,316 are expressed at relatively lower levels in Kc cells than S2 cells (Kc down, shown in green on Fig. 2). While there is a cluster of DEGs expressed at relatively low levels in both cell types (Fig. 2a), there is otherwise a scattered distribution with no clear correlation between expression level in each cell type and the log_2_-fold change in expression between the two cell types (Fig. 2b). Analysis of the top 20 DEGs in each cell type reveals the majority (15 of the 20 Kc up genes and 10 of the 20 Kc down genes) are relatively uncharacterized genes known only by their “CG” designations (Table 3). That so many of the DEGs with the highest log_2_-fold change are uncharacterized is consistent with the idea that each cell line represents a distinct original population of cells that may be relatively rare in the embryo (Chintapalli *et al*. 2007; Leader *et al*. 2018). The fact that “CG” genes are overrepresented in the DEGs also supports the hypothesis that many poorly characterized genes are expressed only in a few cells in the developing embryo (Cherbas *et al*. 2011).

**Table 3.** Top 20 up and down regulated DEGs ranked by log_2_ fold change.

Symbol	Gene ID	KC TPM	S2 TPM	KC Read Count	S2 Read Count	log2 (KC/S2)
**KC UP**						
CG33784	3772181	109.38	0	744	0	13.06
CG33783	3771958	58.52	0	351	0	12.16
CG34269	5740713	56.09	0	467	0	12.10
CG16957	34755	40.58	0	407	0	11.63
CG10339	37854	14.29	0	382	0	10.13
Oatp58Db	37544	12.55	0	358	0	9.94
CG14096	40113	12.13	0	66.41	0	9.89
CG14431	31639	11.52	0.01	869	1	9.82
CG4174	40023	10.14	0	334.06	0	9.63
His1:CG31617	318854	9.45	0	127.11	0	9.53
CG4914	39597	9.33	0	209	0	9.51
CG9672	32651	9.06	0	95	0	9.47
CG5048	39598	9.03	0	114	0	9.47
Oatp58Dc	37545	339.8	0.48	14129	20	9.45
Stl	37619	8.89	0	505	0	9.44
Nerfin-2	41235	7.99	0	269	0	9.29
CG15201	32053	7.95	0	47	0	9.28
CG9297	41688	7.65	0.02	429	1	9.22
GstE1	37106	501.28	0.83	5384	9	9.21
CG5194	39037	33.35	0.07	491	1	9.03
**KC DOWN**						
CG31997	319064	0	141.85	0	1,199	−13.46
CG42369	33894	0	96.87	0	1,827	−12.91
CG12655	33004	0	91.52	0	702	−12.83
CG44013	14462476	0	86.13	0	1,048	−12.74
Dro	36635	0	85.35	0	344	−12.73
l(2)gl	33156	0	68.97	0	5,132	−12.42
Tsp42Eg	35616	0	56.66	0	900	−12.14
CG14273	34146	0	51.43	0	7,24	−12.00
Sp212	2768666	0	47.94	0	1,098	−11.90
Hog	318105	0	41.46	0	318	−11.69
CG34334	5740122	0	38.91	0	1,376	−11.60
CecB	43598	0	38.01	0	191	−11.56
CG43188	12798403	0	37.03	0	381	−11.53
Nolo	35424	0.03	72.51	2	4971	−11.50
p24-2	318890	0	35.58	0	1,656.37	−11.47
CG43088	12797977	0	35.1	0	556	−11.45
PPO1	37044	0.06	163.16	2	5,264	−11.34
CG10026	35226	0	32.48	0	572	−11.34
CG13074	39760	0	30.48	0	634	−11.25
GstZ1	41132	0	30.16	0	361	−11.23

The up regulated genes (expressed at significantly higher levels in Kc cells when compared to S2 cells) and down regulated genes (expressed at significantly higher levels in S2 cells when compared to Kc cells) are listed.

**Fig. 3.**
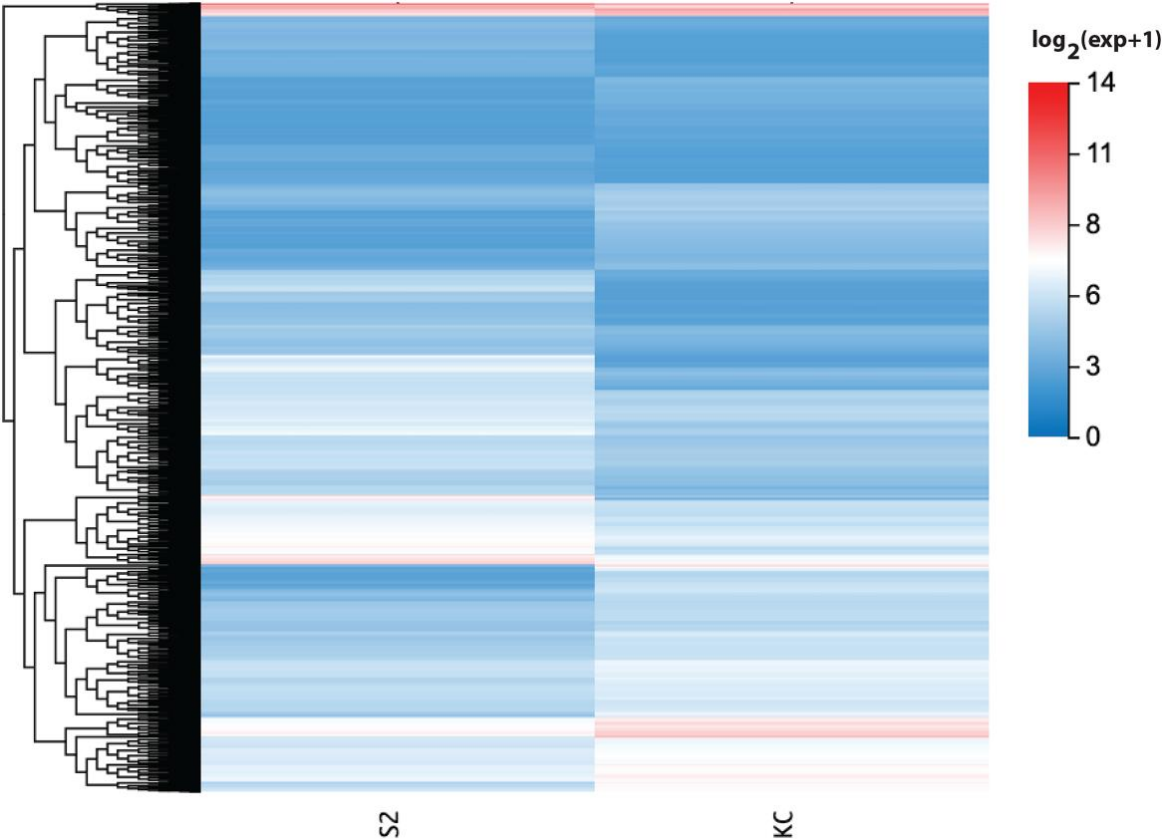
Differentially expressed genes heatmap. The heatmap indicates log_2_ expression level for all 2,588 DEGs in Kc and S2 cells. The color key is shown to the right.

Organizing all 2,588 DEGs in an expression heatmap emphasizes that many, but certainly not all, of the genes are expressed at relatively low levels in the two cell types (Fig. 3). The heatmap also reveals a distinct cluster of seven DEGs that are highly expressed in both cell types (shown in red at the top of Fig. 3). Three of these genes are upregulated *(RpS15Aa*, *CG2493* and *SPARC*) and four are downregulated (*bicaudal*, *CG1943*, *Gapdh2* and *Ino*s). Most of the proteins encoded by the characterized genes in this cluster are involved in well-studied housekeeping cellular functions including translation and glucose homeostasis (Wojtas *et al*. 1992; Park *et al*. 2000). The notable exceptions are *SPARC*, which encodes for a small calcium and growth factor-binding secreted glycoprotein that is synthesized and excreted from hemocytes and enriched in basement membranes (Martinek *et al*. 2008), and *bicaudal*, which encodes the β subunit of the nascent polypeptide-associated complex and is involved in the regulation of *oskar* mRNA localization and *nanos* mRNA translation during the specification of the anterior-posterior axis of the egg (Markesich *et al*. 2000).

**Fig. 4.**
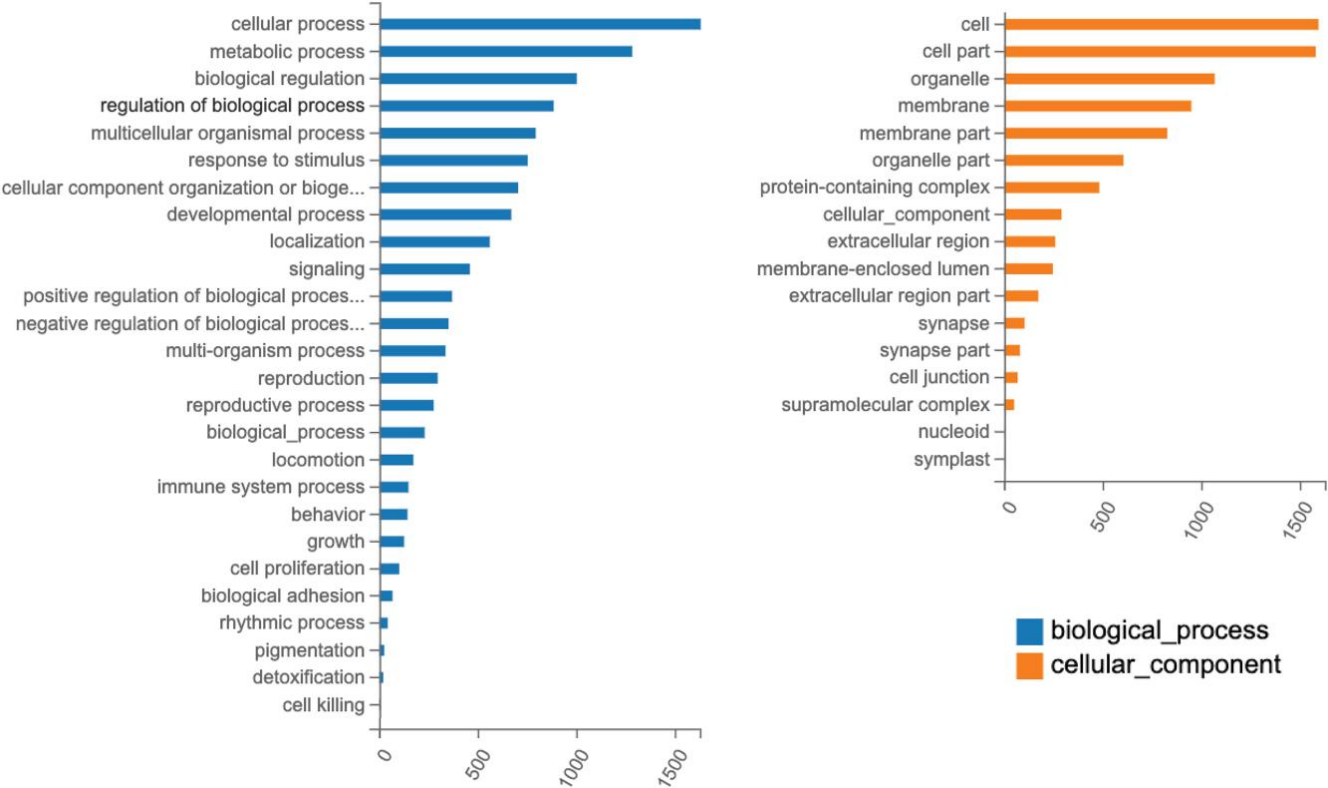
Gene Ontology (GO) classification of DEGs. The number of genes annotated in each functional GO level 2 classification term for; biological process (dark blue), cellular component (orange) and molecular function (light blue) are shown.

Examination of the 2,588 DEGs reveals a wide range of Gene Ontology (GO) terms associated with a variety of cellular structures and functions, such as binding, transport, catalytic activity and transcriptional regulation (Fig. 4). This observation indicates that the DEGs that underlie the molecular differences between the two cell types are not simply restricted to a small subset of biological processes (full list of GO classifications shown in Supplementary Table 8).

### Alternative splicing, SNPs and InDels

**Fig. 5.**
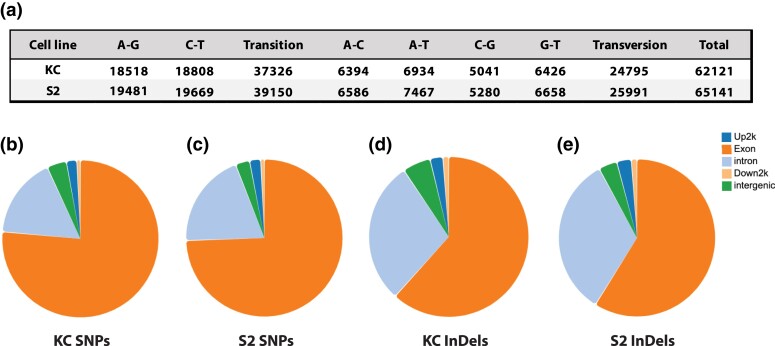
Single Nucleotide Polymorphisms (SNPs) and insertion–deletions (InDels). a) Summary table of all detected SNPs in Kc and S2 cells. The location of the SNPs when mapped on to distinct genomic regions of annotated genes; 5′ upstream 2 kb (Up2k), exons, introns, 3′ downstream 2 kb (Down2k) and intergenic sequences, are shown for Kc b) and S2 c) cells. Similarly, the location of the 7,031 identified InDels in Kc cells d) and 8,066 identified InDels in S2 cells e) are shown.

We also characterized the prevalence of single nucleotide polymorphisms (SNPs), insertion-deletion mutations (InDels) and alternative splicing in the transcriptomes of the two cell types in relation to the annotated *Drosophila* genome. In terms of the SNPs, the overall profile for Kc and S2 cells are very similar, with a total of 62,121 and 65,141 identified SNPs, respectively (Fig. 5a). The SNPs also share a similar distribution when mapped onto gene locations (Fig. 5, b and c). Likewise, the two cell lines harbor a similar number of InDels to each other (7,031 in Kc, 8,066 in S2) with a shared pattern of distribution when mapped to gene locations (Fig. 5, d and e). Perhaps unsurprisingly, a greater proportion of InDels (∼30%) are located in introns when compared to SNPs (∼18%), representing the significant evolutionary pressure to exclude frameshift generating mutations in exons.

Investigation of the alternative splicing landscape in each cell line reveals a similar pattern of distinct splicing events. There are over 1,000 alternative 5′ and 3′ splice sites identified in each cell line, along with over 750 retained introns and over 1,500 skipped exons (Table 4). The only difference of note between the two cell lines is in the number of mutually exclusive exons (MXEs) detected. Such events are defined by the detection of sequencing reads from only one exon in instances where two or more alternate exons are in the annotated gene. When compared to the annotated *Drosophila* genome, 428 MXE events are identified in Kc cells with 1,194 in S2 cells (Table 4). While such events may contribute to the diversity of mRNA transcripts in the two cell types at a functional level, direct comparison between the Kc and S2 cells reveals only 381 distinct alternative splicing events (S2 v KC, Table 4). Considering that expression can be detected for 20,731 distinct mRNAs in at least one of the two cell types, this relatively low number of splicing differences between the entire transcriptomes indicates that the splicing landscape is largely shared. However, this does not exclude the possibility that some of these differences may have important functional consequences for the cells (Venables *et al*. 2012; Mohr and Hartmann 2014).

**Table 4.** Alternative splicing events.

Cell line	A 5′ SS	A 3′ SS	MXE	RI	SE	Total
KC	1,179	1,069	428	794	1,535	5,005
S2	1,241	1,129	1,194	784	1,831	6,179
S2 v KC	74	33	50	81	143	381

The number of distinct alternative splicing events in Kc and S2 cells when compared to the reference *Drosophila* genome is shown. Direct comparison between the two cell types (S2 v Kc) reveals a total of 381 different alternative splicing events, falling in different categories; Alternative 5′ splice site (A 5′ SS), Alternative 3′ splice site (A 3′ SS), Mutually exclusive exon (MXE), Retained intron (RI) and Skipped exon (SE).

## Discussion

Based on our sequencing results, the overall expression profile for the transcriptomes in Kc and S2 cells demonstrate extensive overlap. In Kc cells there was detectable expression from 9,797 different genes and in S2 cells 9,525 genes. These metrics are comparable to the profile in the developing *Drosophila* embryo, in which the number of expressed genes increases from 7,045 in 0–2 h embryos to 12,000 in adult males (Graveley *et al*. 2011). Despite the global similarity of the profile in Kc and S2 cells there are 2,588 identifiable differentially expressed genes (DEGs), indicating that the transcriptional landscape is certainly not identical in the two cell types. To further investigate these differences, and potentially shed light on the origin of the cells and their suitability to study key cellular and molecular processes, we analyzed the expression of three distinct classes of protein-coding genes; early embryonic patterning genes, genes encoding for ligands and receptors in signaling pathways, and genes involved in cellular identity and differentiation during hematopoiesis.

### Early embryonic patterning genes

To investigate if either of the two cell lines retained any features of the transcriptional landscape present in the early *Drosophila* embryo we examined a number of well-characterized genes, most of which encode for transcription factors, responsible for the patterning of the embryo (Wolpert *et al*. 2019). This analysis revealed some key features. Of the 38 genes analyzed, 9 have no detectable expression in either cell type and only 13 have expression at a level >1 TPM in at least one of the cell types (Table 5). This indicates that many of the genes in the early embryonic gene regulatory network are either not expressed or expressed at very low levels (<1 TPM). Closer examination of the genes by classification shows that 8 of the 13 genes with expression at >1 TPM are involved in the patterning of the dorsal–ventral (DV) axis of the early embryo (Table 5). Intriguingly, expression from a similar subset of DV genes was detected in the two cell types, although it should be noted that 7 of the 8 genes demonstrate a significant difference in expression level between the two cell types. In the developing embryo, opposing gradients of *dorsal* (expressed on the ventral side) and *dpp* (expressed on the dorsal side) are critical for patterning across the axis (Sandler and Stathopoulos 2016). In S2 cells, both of these genes are expressed at relatively high levels, along with a number of the downstream genes that the transcription factors encoded by *dorsal* and *dpp* are known to activate including *rho*, *brk*, and *sch* (Hong *et al*. 2008). In Kc cells, a similar subset of DV genes were expressed, but the level of expression for many of the genes is significantly lower (Table 5). Notably, neither *snail* or *twist*, both of which are critical components of the patterning on the ventral side of the developing embryo, are expressed in Kc or S2 cells. Overall, it therefore appears that while expression from most of the genes in the early embryonic gene regulatory network is absent in both cell types, as might be expected for cells isolated from stage 13–15 (Kc) (Cherbas *et al*. 1988; Andres and Cherbas 1992) and stage 16–17 (S2) (Schneider 1972) embryos, some of the critical genes for DV patterning are expressed. This discovery opens up the possibility of potentially utilizing these lines to dissect the regulation of the *Drosophila* DV patterning gene network in a cell-based system. Indeed, through the incorporation of reporter gene systems, it may be feasible to exploit some of the differences in the expression levels of critical DV transcription factors between the two cell lines to perform a detailed dissection of the regulatory network in a tractable and high-throughput manner.

**Table 5.** Expression of early embryonic patterning genes.

Transcription factor	Symbol	Gene ID	Classification	KC TPM	S2 TPM	KC Read Count	S2 Read Count	log2 (KC/S2)
Giant	gt	31227	AP	0.29	2.84	7	69	−3.30
Caudal	cad	35341	AP	1.36	0.03	46	1	5.74
Kruppel	Kr	38012	AP	0	0.03	0	1	−1.00
Dichaete	D	39570	AP	NA	NA	NA	NA	NA
Knirps	kni	40287	AP	0.08	0	2	0	2.58
Huckebein	hkb	40549	AP	0.05	0	1	0	2.00
Bicoid	bcd	40830	AP	4.84	0	168	0	8.57
Hunchback	hb	41032	AP	0.05	0.11	2	5	−1.17
Brinker	brk	31665	DV	25	11.74	1,103	522	1.06
Short gastrulation	sog	32498	DV	0.17	7.59	13	565	−5.43
Decapentaplegic	dpp	33432	DV	0.52	5.34	25	276	−3.37
Mothers against dpp	Mad	33529	DV	14.82	47.07	553	1,775	−1.69
Snail	sna	34908	DV	0.04	0	1	0	1.58
Dorsal	dl	35047	DV	6.44	87.07	253	3,524	−3.78
Schnurri	shn	36171	DV	15.37	31.31	2,010	4,139	−1.05
Twist	twi	37655	DV	NA	NA	NA	NA	NA
Rhomboid	rho	38168	DV	8.69	10.06	304	360	−0.24
Vein	vn	38657	DV	2.31	0.4	164	29	2.49
Zerknult	zen	40828	DV	NA	NA	NA	NA	NA
Tolloid	tld	42945	DV	0.06	0.02	3	1	1.32
Runt	run	33059	PR	0.4	0.7	12	24	−0.81
Sloppy paired 1	slp1	33607	PR	NA	NA	NA	NA	NA
Paired	prd	34629	PR	NA	NA	NA	NA	NA
Hairy	h	38995	PR	251.92	143.98	7,665	4,238	0.78
Fushi-tarazu	ftz	40834	PR	NA	NA	NA	NA	NA
Wingless	wg	34009	SP	0.05	0	2	0	2.00
Patched	ptc	35851	SP	2.29	8.44	176	655	−1.91
Engrailed	en	36240	SP	NA	NA	NA	NA	NA
Hedgehog	hh	42737	SP	0.12	0.18	4	6	−0.58
Frizzled	fz	45307	SP	0.19	0.09	10	5	1.10
Labial	lab	40817	HOX	NA	NA	NA	NA	NA
Proboscidea	pb	40826	HOX	NA	NA	NA	NA	NA
Deformed	Dfd	40832	HOX	0.27	0	10	0	4.39
Sex combs reduced	Scr	40833	HOX	NA	NA	NA	NA	NA
Antennapedia	Antp	40835	HOX	0.04	0	2	0	1.58
Ultrabithorax	Ubx	42034	HOX	0.02	0	1	0	1.00
Abdominal-A	abd-A	42037	HOX	0.04	0	2	0	1.58
Abdominal-B	Abd-B	47763	HOX	0.02	0	1	0	1.00

The read count, TPM values and log_2_ expression ratio for 38 genes known to be expressed in the early embryonic patterning gene regulatory network are shown for the two cell types. The genes are functionally sub-categorized as anterior–posterior axis specification (AP), dorsal–ventral axis specification (DV), pair-rule (PR), segment-polarity (SP) or homeotic (HOX) genes. NA indicates no expression was detected in either Kc or S2 cells.

### Signaling pathways

**Fig. 6.**
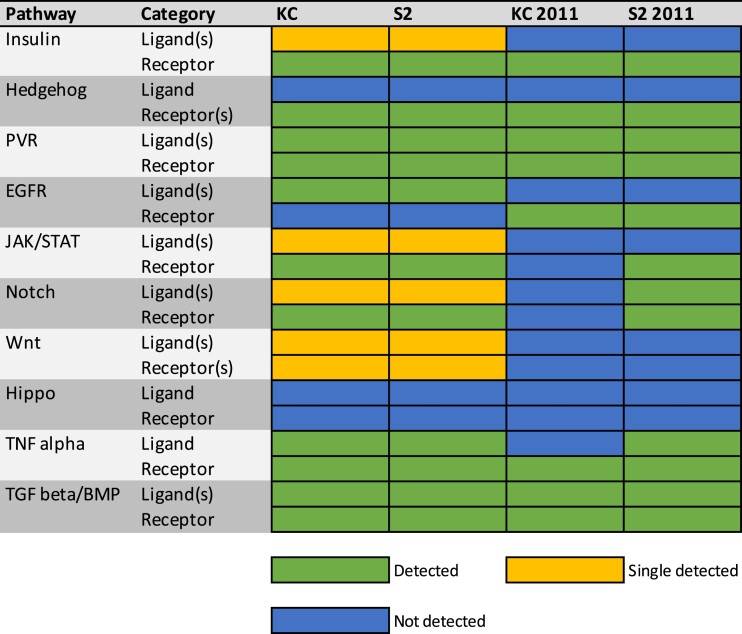
Summary expression of signaling pathway ligands and receptors. The expression profile for the ligand(s) and receptor(s) in 10 different signaling pathways in the two cell types are shown, along with the previously reported microarray expression profile (Cherbas *et al*. 2011). Color coding indicates expression was detected (green), not detected (blue), or, in the case where multiple ligands or receptors exist, expression of a single ligand or receptor was detected (yellow).

We analyzed the expression in both cell types of 10 signaling pathways: Insulin, Hedgehog, PVR, EGFR, JAK/STAT, Notch, Wnt, Hippo, TNF alpha, and TGF beta/BMP. For each pathway, we examined the expression levels of known ligands and receptors (Supplementary Table 9). The expression patterns are summarized in Fig. 6 and indicate that the signaling landscape is extensively shared between the two cell types, with seven of the 10 pathways active. Insulin signaling is on, but predominantly mediated through the Insulin-like peptide 6 (Ilp6) ligand. Hedgehog is off, as the ligand is absent. PVR is on, while EGFR is off due to the very-low expression of the EGFR receptor. JAK/STAT is on, but with relatively low levels of the ligands. Notch is on, with detectable levels of the Serrate (Ser) ligand, but not the Delta ligand. Wnt is on, but predominately restricted to the Wnt5 ligand and Frizzled 4 (fz4) receptor. Hippo is off, while TNF alpha and TGF beta/BMP signaling are on.

These results largely align with the prior detailed characterization of these pathways (Cherbas *et al*. 2011), but there are some potentially intriguing differences. While a number of the more subtle differences observed can likely be attributed to the higher level sensitivity in our expression analysis that is not true in every case. For example, in the EGFR pathway expression of the canonical EGFR receptor was detectable in the previous analysis (Cherbas *et al*. 2011), but it appears to be absent in Kc cells and expressed at very low levels (TPM 0.62) in S2 cells (Supplementary Table 9) in our current study. The Wnt pathway is a further example of deviation between the two studies, as the fz4 receptor and Wnt5 ligand are both detectable in Kc and S2 cells in our work, while their expression was reported as absent in the earlier study (Cherbas *et al*. 2011). Such differences could be accounted for by the inherent variation found in cell lines cultured in different laboratories, in this particular case over a decade apart, but may warrant further investigation to reveal the extent to which differences in the signaling pathways are potentially dynamic over time. Despite these differences, the confirmed widespread expression of the molecular components of many of the signaling pathways in these cells indicates that they will continue to be a valuable tool to study *Drosophila* cell–cell interactions in future studies.

### Hematopoietic origins

**Fig. 7.**
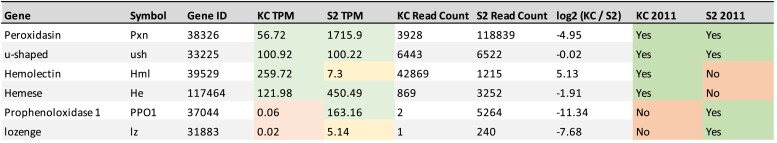
Expression of hematopoietic marker genes. The read count, TPM values and log_2_ expression ratio for six hemocyte marker genes in the two cell types are shown along with the previously reported microarray expression profile (Cherbas *et al*. 2011). Color coding indicates TPM expression level as very low (<1, red), low (1–10, yellow) or high (>10, green).

In order to investigate the potential hemocyte or hematopoietic origin of the two cell lines, we analyzed expression of gene markers from these lineages (Fig. 7). In *Drosophila*, three distinct types of hemocytes originate from a common precursor stem-cell like population: plasmatocytes, crystal cells, and lamellocytes (Lebestky *et al*. 2000). Previous studies have indicated that Kc cells have a plasmatocyte identity and that S2 cells combine some properties of plasmatocyte and crystal cells, based on gene expression patterns measured using microarrays (Cherbas *et al*. 2011). Specifically, both cell types were found to express the plasmatocyte marker *Pxn* along with *ush*, an inhibitor of crystal cell differentiation (Fossett *et al*. 2001). In our RNA-seq data, we also detect high levels of these two genes in Kc and S2 cells (Fig. 7). The more general hemocyte markers *Hml* (Charroux and Royet 2009) and *He* (Lebestky *et al*. 2000) are also detectable in both cell types in our data, which contrasts with the failure to detect expression of these two genes in S2 cells in the earlier study (Cherbas *et al*. 2011). This discrepancy could, in part, be due to the increased detection sensitivity in our current study, although the level of *He* expression (TPM 450.49) we detect is relatively very high. S2 cells do express a high level of *PPO1* and a detectable level of *lz*, both of which are associated with crystal cells and not with plasmatocytes (Jacques *et al*. 2009; Yu *et al*. 2018; Koranteng *et al*. 2020). Expression of these two crystal cell markers is absent in Kc cells, in agreement with the earlier microarray analysis (Fig. 7). In summary, our new data confirm the conclusion that both cells have a hematopoietic origin, but that the lines are distinct from each other. Kc cells appear to have a clear plasmatocyte identity, while the S2 cells express markers of both plasmatocytes and crystal cells and therefore may represent a certain level of transcriptional plasticity not seen in any particular hemocyte cell type found in the embryo. Further study will be required to clarify the detailed molecular identity of the S2 cells, including single cell transcriptome analysis to investigate the possibility of a mixed cell population.

## Supplementary Material

jkad054_Supplementary_Data

## Data Availability

The datasets supporting the results of this article are available at the NCBI Sequence Read Archive (SRA) under BioProject accession number PRJNA937779. Supplemental material available at G3 online.
