# Supplementary material for: Transcriptome profile in Drosophila Kc and S2 embryonic cell lines
Source: G3 (Bethesda). 2023 Mar 3;13(5):jkad054. doi: 10.1093/g3journal/jkad054 (PMC10151398; doi:10.1093/g3journal/jkad054)
Supplement: jkad054_Supplementary_Data [file jkad054_supplementary_data.zip › Supplemental_Material_Legends_G3-2023-404112.docx]

**Supplementary Information**

**S1 Table. All 20,731 annotated mRNA transcripts with detectable expression in either KC and/or S2 cells.**

The RNA ID, annotated genomic location, read count and calculated TPM and FPKM expression values for Kc and S2 cells are shown along with the log_2_ expression ratio, *P* value and FDR value for comparing expression between Kc and S2 cells. The associated functional Gene Ontology (GO) level 2 classification term(s) for cellular component (GO_C), molecular function (GO_F) and biological process (GO_P) are indicated for each annotated transcript.

**S2 Table. All 10,554 annotated genes with detectable expression in either KC and/or S2 cells.**

The gene symbol, annotated genomic location, read count and calculated TPM and FPKM expression values for Kc and S2 cells are shown along with the log_2_ expression ratio, *P* value and FDR value for comparing expression between Kc and S2 cells. The associated functional Gene Ontology (GO) level 2 classification term(s) for cellular component (GO_C), molecular function (GO_F) and biological process (GO_P) are indicated for each annotated gene.

**S3 Table. All 985 Kc-only expressed genes.**

The read count, TPM values and log_2_ expression ratio for the 985 genes with expression only in Kc cells are shown for the two cell types.

**S4 Table. All 723 S2-only expressed genes.**

The read count, TPM values and log_2_ expression ratio for the 723 genes with expression only in S2 cells are shown for the two cell types.

**S5 Table. Top 20 genes with the highest detectable expression in Kc cells ranked by TPM.**

The read count, TPM values and log_2_ expression ratio are shown for Kc and S2 cell types.

**S6 Table. Top 20 genes with the highest detectable expression in S2 cells ranked by TPM.**

The read count, TPM values and log_2_ expression ratio are shown for Kc and S2 cell types.

**S7 Table. All 2588 Differentially Expressed Genes (DEGs) when comparing KC and S2 cells.**

The read count, TPM values and log_2_ expression ratio are shown for Kc and S2 cell types. The DEGs are ranked by log_2_ expression ratio. Genes with a log_2_ expression ratio < 1 and > -1 (i.e. less than a two-fold change) are considered non-DEGs.

**S8 Table. GO classification of all 2395 DEGs with an annotated term.**

The associated functional Gene Ontology (GO) level 2 classification term(s) for cellular component, molecular function and biological process are indicated for each annotated DEG.

**S9 Table. Expression of signaling pathways.**

The read count, TPM values and log_2_ expression ratio for the ligands and receptors in 10 different signaling pathways are shown for the two cell types. Expression TPM values greater than 1 are indicated in green.
